# β-Cyclodextrin Does not Alter the Bioaccessibility and the Uptake by Caco-2 Cells of Olive By-Product Phenolic Compounds

**DOI:** 10.3390/nu10111653

**Published:** 2018-11-03

**Authors:** Aurélia Malapert, Valérie Tomao, Marielle Margier, Marion Nowicki, Béatrice Gleize, Olivier Dangles, Emmanuelle Reboul

**Affiliations:** 1 Avignon University, INRA, UMR408 SQPOV, F-84000 Avignon, France; malapert.aurelia@gmail.com (A.M.); Valérie.Toma@univ-avignon.fr (V.T.); Beatrice.Gleize@inra.fr (B.G.); Olivier.Dangles@univ-avignon.fr (O.D.); 2 Aix-Marseille University, INRA, INSERM, C2VN, 13385 Marseille, France; Marielle.Margier@univ-amu.fr (M.M.); Marion.Nowicki@univ-amu.fr (M.N.)

**Keywords:** hydroxytyrosol-*O*-glucoside, tyrosol, caffeic acid, *p*-coumaric acid, alperujo, olive pomace, polyphenols, bioavailability, in vitro digestion, enterocytes

## Abstract

Alperujo—a two-phase olive mill waste that is composed of olive vegetation water and solid skin, pulp, and seed fragments - is a highly valuable olive by-product due to its high content in phenolic compounds. In this study, we assessed whether β-cyclodextrin (β-CD), which is used to extract and protect alpejuro phenolic compounds (hydroxytyrosol-*O*-glucoside, tyrosol, caffeic, and *p*-coumaric acids) could impact on their bioaccessibility (i.e., the percentage of molecule found in the aqueous phase of the digesta) and uptake by intestinal cells, by using an in vitro digestion model and Caco-2 TC7 cells in culture, respectively. Our results showed that β-CD did not change the bioaccessibility of the selected phenols. Hydroxytyrosol-*O*-glucoside and caffeic did not cross Caco-2 cell monolayers. Conversely ferulic acid, identified as the main caffeic acid intestinal metabolite, was absorbed through intestinal cell monolayers (~20%). Interestingly, β-CD moderately but significantly improved the local absorption of tyrosol and *p*-coumaric acid (2.3 + 1.4% and 8.5 ± 4.2%, respectively, *p* < 0.05), even if their final bioavailability (expressed as bioaccessibility × absorption by Caco-2 cells) was not modified (16.2 ± 0.6% vs. 16.8 ± 0.5% for tyrosol and 32.0 ± 3.2% vs. 37.2 ± 3.2% for *p*-coumaric acid, from pure alperujo and alperujo complexed with β-CD, respectively). Overall, our results show that β-CD is an interesting extraction and storage agent for phenolic compounds that does not alter their in vitro bioavailability.

## 1. Introduction

About 98% of the global olive production originates from the Mediterranean basin, among which 73% from Southern Europe with Spain, Italy, and Greece as the main producers [1,2]. Alperujo is a two-phase olive mill waste that is composed of olive vegetation water and solid skin, pulp, and seed fragments [3]. Because of its high moisture and high phenolic content, this semi-solid leftover is considered to be one of the most abundant Mediterranean pollutants [4].

**Figure 1:**
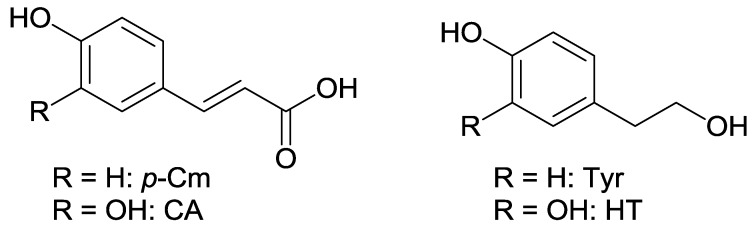
Chemical structures of the selected olive phenols. In hydroxytyrosol-glucosides (HT-Glc), the Glc unit can be attached to any of the three OH groups of HT.

Many studies confirmed the potential of alperujo as a concentrated and cost-effective source of olive phenolic compounds. Phenyl alcohols and cinnamic acids - including hydroxytyrosol (HT), tyrosol (Tyr), caffeic acid (CA), and *p*-coumaric acid (*p*-Cm) and their glycosides, such as HT-*O*-glucosides (HT-Glc) are the main phenolic classes found in this olive pomace [5,6] (Figure 1).

These biophenols have been associated with health benefits, such as anti-inflammatory, antidiabetic, cardioprotective, and anti-apoptotic activities, owing to their ability to reduce oxidative stress and improve the antioxidant status of individuals [7]. Thus, they have a high potential of valorization in the food, cosmetic, and pharmaceutical industries.

Cyclodextrins (CDs) are cyclic oligosaccharides composed of glucopyranose units that are connected by α-1,4 glycosidic bonds. All OH groups point to the exterior of the cavity, thus conferring water solubility to CDs. However, the CD cavity, which is lined by CH groups, is more hydrophobic, so that CDs can act as encapsulation agents for a large variety of relatively non-polar compounds that are able to enter the cavity [8,9]. Composed of seven d-glucose units, β-cyclodextrin (β-CD) is the most common CD due to its low price, its safety, and its efficiency at forming inclusion complexes with a wide variety of molecules displaying a MM < 800 g/mol [10]. CDs can enhance the extraction of phenolic compounds from plant material [11]. CDs can thus be used as sustainable phenolic compound extraction agents. Furthermore, CD-bound phenolic compounds are more stable [12,13], which can be useful during extraction and storage over long periods [14,15,16]. The bioavailability of phenolic compounds may also be enhanced in the presence of CDs, as CDs can improve their water solubility [17,18].

HT bioavailability has been studied in detail in several studies [19,20,21,22]. In particular, we previously showed that β-CD did not alter HT in vitro bioavailability [23]. However, the effect of β-CD on other phenolic compound bioavailability remains unknown. In this study, we thus focused on four major phenolic compounds from alperujo (Aglandau variety), i.e., HT-Glc, Tyr, CA and *p*-Cm, and assessed i) their fate during the digestion process and ii) their absorption by enterocytes, in the presence or absence of β-CD, by using an in vitro digestion model and a human intestinal cell model in culture, respectively.

## 2. Materials and Methods

### 2.1. Chemicals

β-CD was from Roquette Frères (Lestrem, France). Hydroxytyrosol was a generous gift from Pr Visioli (Madrid, Spain). Gallic acid, tyrosol, caffeic acid, *p*-coumaric acid, pepsin, porcine pancreatin, porcine bile extract, formic acid, water, ethanol, and acetonitrile were from Sigma-Aldrich (Saint-Quentin Fallavier, France). Dulbecco’s modified Eagle’s medium (DMEM) containing 4.5 g/L glucose, non-essential amino-acids, penicillin/streptomycin, trypsin-EDTA (500 mg/L and 200 mg/L, respectively), phosphate-buffered saline (PBS), and Hanks’ balanced salt solution (HBSS) were purchased from Life Technologies (Illkirch, France). Fetal bovine serum was from PAA (Vélizy Villacoublay, France). Alperujo was collected from a two-phase centrifuge mill (Moulin Castelas, Baux-de-Provence, France). Foods were purchased from a local Casino supermarket (Marseille, France).

### 2.2. Preparation of Alperujo Sample

Alperujo (72% of moisture) contained in cheesecloth canvas was pressed and successively filtered through celite and through 0.45 µm and 0.2 µm filters (VWR, Radnor, PA, USA). Proteins were removed by ethanol precipitation (42%). Ethanol was then evaporated and the aqueous alperujo fraction was frozen at −20 °C.

### 2.3. Preparation of Inclusion Complexes with β-CD

The protein-free alperujo aqueous fraction was diluted to reach a total phenolic content of 5 mM in gallic acid equivalent. β-CD was then added to a 1:1 molar ratio and the solution stirred for 1 h at room temperature. After freeze-drying, the alperujo-CD powder was stored at −20 °C in amber glass. As a control, a freeze-dried powder of aqueous alperujo fraction without β-CD was also prepared.

### 2.4. In Vitro Digestions

Alperujo samples, complexed or not with β-CD, were added to test meal composed of 6.7 g of pureed potatoes, 1.2 g of fried minced beef, and 0.2 g of refined olive oil. The final amounts of phenolic compounds in the meal were: 9.7 mg of HT-Glc, 12.3 mg of Tyr, 0.8 mg of CA, and 0.5 mg of *p*-Cm. The in vitro digestion was carried out as previously described [23,24]. Briefly, meal components (including phenolic samples) were mixed with 32 mL of 0.9% NaCl. The mixture was dispersed with Ultra-Turrax^®^ (Ika) and artificial saliva (2.5 mL, pH 7) was added to the mixture. The sample was incubated for 10 min at 37 °C in a shaking incubator. The pH of the gastric medium was adjusted to 4.00 ± 0.02 with 1 M HCl. Then, porcine pepsin (2 mL, 40 mg/mL in 0.1 M HCl) was added and the homogenate was incubated at 37 °C in a shaking incubator for 30 min simulating the gastric step. The pH of the mixture was raised to 6.00 ± 0.02 with 0.9 M NaHCO_3_ and 13 mL of a mixture of porcine bile extract (39.08 mg/mL) and pancreatin (2.08 mg/mL) in 0.1 M trisodium citrate (pH 6) were added. Samples were incubated in a shaking incubator at 37 °C for 30 min to complete the digestion process. The final mixture representing the digesta was centrifuged (2500 g for 1 h at 10 °C) to separate the aqueous fraction. Finally, the aqueous fraction was successively passed through 0.8 µm and 0.2 µm filters (Millipore, Burlington, MA, USA).

All analyses were run in quadruplicate. Aliquots from salivary, gastric, and duodenal steps were frozen at −80 °C until use.

### 2.5. Cell Experiments

Caco-2 TC7 cells were grown in DMEM supplemented with 16% (v/v) fetal bovine serum, 1% (v/v) non-essential amino-acids, and 1% (v/v) antibiotics (complete medium), as previously described [25]. Cells were then seeded at a density of 15 000 cells/well, grown on transwell membrane (six-well plate, 1 mm pore size polycarbonate membrane; Becton Dickinson) and incubated in humidified atmosphere of 5% CO_2_ and 95% air at 37 °C during 21 days to obtain confluent and differentiated cell monolayers.

The cytotoxicity on Caco-2 cells of the aqueous fractions from in vitro digestions was first assessed to determine the suitable dilution required before adding the aqueous fractions to the apical side of cell monolayers. These preliminary results showed that a 1/10 dilution in the culture medium was necessary. To avoid any interference with DMEM or serum components, the aqueous fractions were diluted in HBSS. Caco-2 cells also received HBSS in both chambers 12 h before the experiments. At the beginning of each experiment, cells were rinsed twice with 1 mL of PBS. Then, 1 mL of diluted aqueous fraction was added to the apical side and 2 mL of HBSS were added at the basolateral side. After an incubation period (2, 4, or 6 h) at 37 °C, both apical and basolateral media were harvested. Cells were then rinsed twice with 1mL of PBS and scrapped in 0.5 mL of iced-cold PBS. All analyses were run in quadruplicate and samples were stored at −80 °C until further analysis.

### 2.6. Analyses

#### 2.6.1. Extraction of Digestion and Cell Media Samples

Gallic acid was chosen as internal standard as it was not present in the extract [23]. Phenolic compounds were extracted from salivary, gastric and duodenal samples as follows: 0.3 mL of ethanol and 0.2 mL of n-hexane were added to 0.2 mL of sample and the mixture was stirred for 10 min using a vortex blender at maximal speed. After centrifugation (3560 g for 10 min at 4 °C), the lower phase was collected. The solid bottom was re-extracted with 0.3 mL of ethanol and vortexed for 10 min. The second ethanol phase recovered was pooled to the previously collected phase, and then dried using a Speed-Vac^®^. The dried extracts were dissolved in 200 µL of water. Apical media were directly injected. To concentrate them, basolateral media (1900 µL) were dried using a Speed-Vac^®^, and the dried residues were dissolved into 80 µL of water. All samples were stored at −80 °C before analysis.

#### 2.6.2. Extraction of Cell Samples

Following the procedure by Gallardo et al. (2016) [21], 50 µL of internal standard were added to harvested cells (in 500 µL PBS). Samples were then sonicated for 10 min at room temperature and centrifuged (10,000 g for 10 min at 4 °C) [21]. Supernatants were collected and evaporated to dryness. The dried residues were dissolved in 50 µL of water and stored at −80 °C before analysis.

#### 2.6.3. Chromatographic Analysis

All samples were analyzed by UHPLC-DAD-MS using an Acquity UPLC^®^ system that was linked to both a diode-array detector and a Bruker Daltonics HCT Ultra Ion Traps mass spectrometer equipped with an Electron Spray Ionization (ESI) source operating in negative ion mode. The separation was performed on an Acquity C18 BEH column (50 × 2.1 mm i.d., 1.7 µm) kept at 35 °C. The solvents were (A) water/formic acid (99.5/0.5) and (B) acetonitrile. The proportions of solvent B used were: 0–10 min: 1–20%, 10–12 min: 20–30%, 12–14 min: 30–100%. Along the three steps of the gradient, the flow rate was 0.30, 0.35, and 0.40 mL/min. The injection volume was 1 μL for digestion and apical medium samples, and 10 µL for cells and basolateral samples. Chromatograms were acquired at 280 nm. The spectroscopic detection was performed in the range 200–800 nm with a resolution of 1.2 nm. The concentrations of phenolic compounds Tyr, CA, and *p*-Cm were estimated from calibration curves (peak area vs. concentration) with R² values greater than 0.99 [26].

#### 2.6.4. Mass Spectrometry

ESI mass spectra were obtained at ionization energies of 50 and 100 eV (capillary voltage = 2 kV, source temperature = 365 °C, skimmer voltage = 40 V). The drying gas was introduced at a flow rate of 10 L/min. Scans were performed in the *m*/*z* range 100–2000.

### 2.7. Statistical Analyses

Results were expressed as means ± standard deviations. Differences between means were assessed using ANOVA followed by the post-hoc Tukey test for parametric data. P values under 0.05 were considered to be significant. Two parameters were assessed to express the bioavailability: a) the ratio between the mass of phenolic compounds recovered at the basolateral side and the initial mass added to the apical side, b) the ratio between the mass of phenolic compounds recovered at the basolateral side and the initial mass added to the meal.

## 3. Results

### 3.1. Bioaccessibility of the Phenolic Compounds

**Figure 2:**
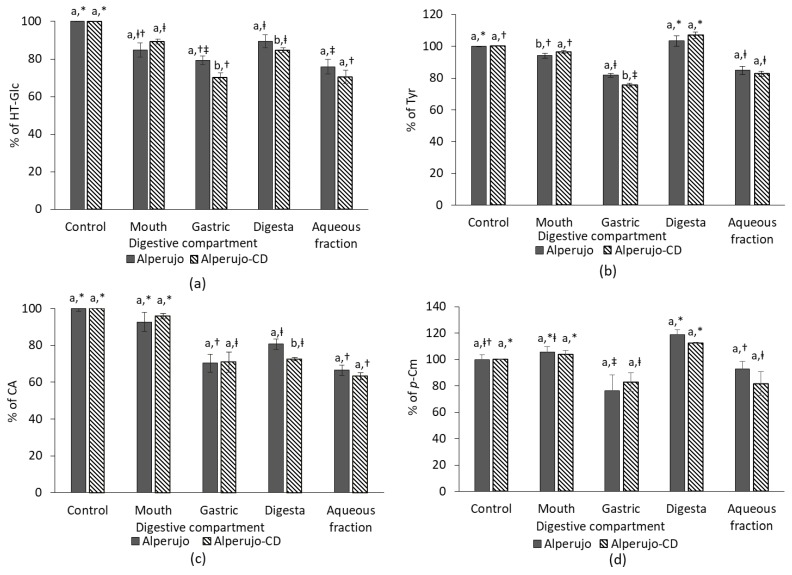
Bioaccessibility of phenolic compounds from alperujo powders in each digestive compartment. (**a**) HT-Glc; (**b**) tyrosol (Tyr); (**c**) caffeic acid (CA); and, (**d**) *p*-coumaric acid (*p*-Cm) from either pure alperujo (grey bars) of alperujo complexed with β-CD (alperujo-CD, dashed bars). Values are expressed as mean ± SD (*n* = 4). Different letters indicate a significant difference according to Tukey test (*p* ≤ 0.05) between alperujo and alperujo-CD conditions in each compartment. Different symbols indicate a significant difference according to Tukey test (*p* ≤ 0.05) between the compartments for each condition (alperujo or alperujo-CD).

Figure 2 shows the bioaccessibility of HT-Glc, Tyr, CA, and *p*-Cm from alperujo, complexed, or not with β-CD, in each digestive compartment during the in vitro digestion process.

The presence of β-CD has no effect in most compartments during the digestion process of HT-Glc, Tyr, CA, and *p*-Cm. Some small but significant differences have been observed regarding the recovery of HT-Glc in the gastric and the digesta fractions, of Tyr in the gastric compartment and of CA in the digesta. However, no effect of β-CD has been observed on the final bioaccessibility of all studied phenolic compounds (Figure 2). On average, HT-Glc concentration decreased during the digestion process to reach a bioaccessibility of 73.2 ± 4.5%. 83.7 ± 2.4% of Tyr was recovered in the final aqueous phase at the duodenal step. Like Tyr, *p*-Cm, which is a monophenol, was highly soluble in the aqueous phase and was recovered at more than 87.9 ± 7.5 %, while CA recovery was about 64.9 ± 2.9%. The amounts of Tyr and *p*-Cm recovered in digestas seemed to be higher than the initial quantity (Tyr from alperujo-CD and *p*-Cm from alperujo, *p* < 0.05).

### 3.2. Uptake of the Phenolic Compounds by Caco-2 TC7 Cells

The ability of HT-Glc, Tyr, CA and *p*-Cm to cross the intestinal barrier was evaluated in Caco-2 cells using the previous aqueous fractions from in vitro digestions. According to preliminary cytotoxicity assays, a 1:10 dilution of the aqueous fractions was required to perform the study.

**Figure 3:**
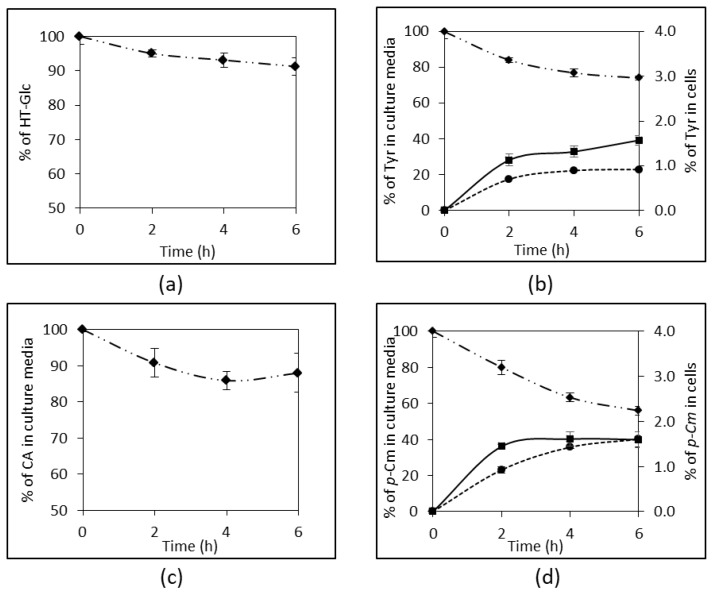
Recovery of the phenolic compounds in both the apical and basolateral chambers, and in Caco-2 cell compartment. (**a**) HT-Glc; (**b**) Tyr; (**c**) CA; and, (**d**) *p*-Cm. All results are expressed as percentage of the initial amount at the apical side. Values are expressed as mean ± SD (*n* = 4). ◆: apical side; ●: basolateral side; ■: Caco-2 cells.

Our results showed that each phenolic compound exhibited the same absorption profile whether it was from alperujo or from alperujo-CD powder. Therefore, Figure 3 presents the recovery of the phenolic compounds from alperujo only.

After a 6-h incubation period on cell monolayers, a decrease of HT-Glc and CA amounts of about 10% (*p* < 0.05) and 18% (*p* < 0.052), respectively, was measured in the apical media (Figure 3a,c). However, neither HT-Glc nor CA was recovered in the cytosolic compartment of the cells or in the basolateral media.

Conversely, both Tyr and *p*-Cm were found in the cytosolic compartment of Caco-2 cells after a 6h-incubation period: 1.6 ± 0.1% vs. 1.4 ± 0.1% = for Tyr, and 1.6 ± 0.1% vs. 1.8 ± 0.2% = for *p*-Cm, from alperujo (Figure 3b,d) vs. alperujo-CD; respectively. Both phenols were recovered in much higher amounts on the basolateral side after 6 h. Interestingly, the presence of β-CD slightly improved membrane crossing, i.e., 22.9 ± 0.5% vs. 25.2 ± 1.3% for Tyr (*p* < 0.022), and 40.2 ± 4.1% vs. 48.7 ± 2.0%for *p*-Cm (*p* < 0.021) from alperujo (Figure 3b,d) vs. alperujo-CD, respectively. Our data also showed that the absorption of Tyr and *p*-Cm into Caco-2 cells and their passage to the basolateral side were time-dependent. Moreover, the total recovery of Tyr and *p*-Cm in these three compartments was nearly 100%, suggesting that no metabolism occurred in the intestinal cells with these two monophenols.

**Figure 4:**
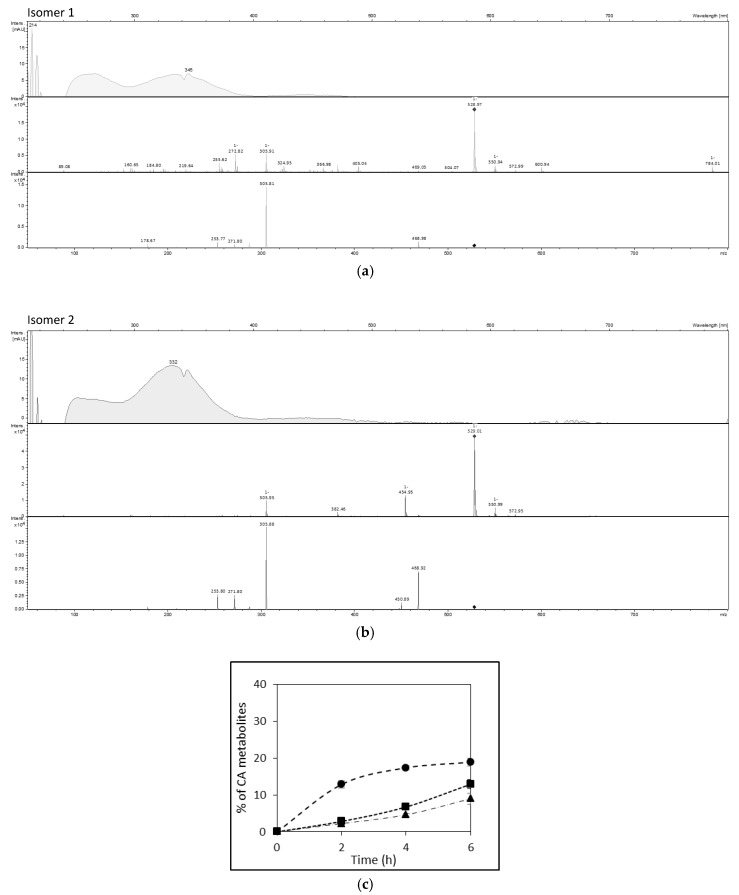
(**a**,**b**) UHPLC-DAD-MS analysis of caffeic acid metabolites. UV-visible, MS1 and MS2 data of two isomers tentatively identified as oxidized CA trimers (CA3: *m*/*z* = 3 × 180 − 4 − 1 = 535, oxidized CA3 (CA3-6H): *m*/*z* = 536 – 6 − 1 = 529). (**c**) Metabolites of CA from alperujo in culture media (B). Values are expressed in CA equivalent as mean ± SD (*n* = 4). ●: FA in the basolateral side; ■: isomer 1 of CA metabolite in cells; ▲: isomer 2 of CA metabolite in cells.

By contrast, three metabolites of CA were identified in culture media. Ferulic acid (FA), formed by O-methylation at C3-OH, was identified by its molecular ion at *m*/*z* 193 and its UV spectrum, and by comparison with a FA standard. Two other CA metabolites were also detected. They shared the same UHPLC-DAD-MS characteristics: a molecular ion at *m*/*z* 529, an intense fragment at *m*/*z* 306, a UV absorption band in the range 320–340 nm characteristic of hydroxycinnamic acids, and a weak absorption band in the visible range (420–480 nm) (Figure 4a,b). These two isomers are proposed to be oxidized CA trimers (trimmers-6H) (see Discussion section).

FA found at the basolateral side represented 18.8 ± 1.0% and 23.4 ± 2.2% (CA equivalent) of the initial CA amount in the apical side, from alperujo and alperujo-CD, respectively (*p* < 0.009). The two other CA derivatives found into the cells represented 12.9 ± 1.3% and 9.0 ± 1.5% of initial CA, for isomers 1 and 2, respectively (Figure 4c). Taking into account the presence of the three metabolites, the total recovery of CA was 128.9 ± 4.2% (*p* < 0.0001).

### 3.3. Overall Bioavailability of the Phenolic Compounds

The in vitro bioavailability has been firstly calculated taking into account the phenolic amount recovered in the basolateral compartment as compared to the initial amount provided at the apical side of the cultured cells (Table 1). *p*-Cm displayed a bioavailability higher than 40% whether it was from alperujo or alperujo-CD. Intact CA was not recovered, while FA, its *O*-methylether, showed an absorption efficiency of about 20% of the initial CA amount. Tyr also displayed a bioavailability higher than 20%. In all cases, the phenolic compounds from alperujo-CD were better absorbed than from alperujo (*p* < 0.05 for Tyr and *p*-Cm from alperujo and alperujo-CD, respectively).

The bioavailability was also assessed by comparing the Tyr, CA, *p*-Cm, and FA contents at the basolateral side of cell monolayers with the initial amounts of phenols provided in the test meal before in vitro digestion (Table 2). With these calculations, no significant differences were found between the phenolic compounds whether they were from alperujo or alperujo-CD.

## 4. Discussion

This study explored the in vitro bioaccessibility and bioavailability of HT-Glc, Tyr, CA, and *p*-Cm from alperujo powders. In presence of β-CD, the bioaccessibility of HT-glc, Tyr and CA appeared to be slightly lower in some digestive compartments. As β-CD precipitates with ethanol, this may be due to a loss of a part of the complexed phenolic compounds during sample extraction. However, no effect of β-CD was observed on the final bioaccessibility of phenolic compounds from alperujo powders. The amounts of each phenol decreased during the digestion process to reach an average aqueous bioaccessibility of 73.2 ± 4.5%, 83.7 ± 2.4%, 64.9 ± 2.9%, and 87.9 ± 7.5% for HT-Glc, Tyr, CA, and *p*-Cm, respectively. Our results highlight that *p*-Cm is more bioaccessible than CA (+25%). D’Antuono et al. (2016) also observed that Tyr from olives exhibited a higher bioaccessibility (+16%) than HT [27]. These results confirm that monophenols, like *p*-Cm and Tyr, are more stable during digestion than their ortho-diphenol homologues, i.e., CA and HT, respectively. This is consistent with the well-known sensitivity of catechols to autoxidation [28]. The recovery of *p*-Cm and Tyr in the digesta reached or exceeded 100%, which probably reflects the hydrolysis of other phenolic compounds from alperujo samples, resulting in both Tyr and *p*-Cm release. For instance, an apparent decrease of comselogoside was observed in our experiment, which could be due to to hydrolysis into *p*-Cm.

The Caco-2 TC7 cell model was then chosen to monitor phenolic compound absorption [29,30]. The apical to basolateral transport of Tyr through Caco-2 cells reached almost 22.9 ± 0.7% of its initial apical content in six hours. Furthermore, it was also recovered in the cellular compartment. Consistently, with the results of Corona et al. (2009), no metabolization was evidenced in Caco-2 cells [31]. Despite the time-dependent decrease of the amount of HT-Glc in the apical chamber, no HT-Glc was recovered in cells or in the basolateral chamber. β-d-Glucosides of dietary plant phenols are resistant to acid hydrolysis at the gastric level and reach the intestine in their intact form. In general, they are not or only slightly absorbed because of their polarity and size, while aglycones, released by the action of intestinal β-glucosidase, can be absorbed [32,33]. Manna et al. (2000) observed that HT was absorbed by passive diffusion [19]. Besides, a direct transport through the Na^+^-dependent d-glucose transporter was evidenced as a minor absorption route for quercetin O-β-d-glucosides [30], which may also operate for HT-Glc. In our model, CA was not found bioavailable in its native form, whereas *p*-Cm was highly absorbed and transported through Caco-2 cells, even though its initial amount was lower. The bioavailability of poorly water-soluble CA is known to be low [34,35]. Konishi et al. (2004) showed that CA absorption (5 mM CA solution at the apical side) was more efficient when a proton gradient is established (apical: pH 6; basolateral: pH 7.4) [36]. Tsukagoshi et al. (2007) compared the uptake by Caco-2 cells of hydroxybenzoic and hydroxycinnamic acids and observed that dihydroxylation decreased drastically their uptake by cells. Dihydroxylation may decrease the affinity for the monocarboxylic acid transporters (MCTs) that are involved in the absorption of phenolic acids [37]. Unlike *p*-Cm, CA undergoes extensive metabolism upon absorption. Our results revealed that the total recovery of CA and its metabolites exceeded 100%. FA, which results from catechol-*O*-methyltransferase (COMT) activity, and two isomeric metabolites were evidenced. These two isomers are proposed to be oxidized CA trimers (trimers – 6H) by analogy with oxidized dimers of CA derivatives already detected in apple juices and ciders [38]. Their presence in cells suggests that these metabolites were formed by intracellular enzymatic oxidation of CA. Possible peroxidase activity in Caco-2 cells was already shown to oxidize quercetin, a first step toward covalent coupling with cell proteins [39]. The mass excess probably reflects the metabolism of CA derivatives that are naturally found in alperujo like caffeoyl-6′-secologanoside, previously identified in our alperujo samples [26].

Tyr, *p*-Cm and CA from alperujo-CD were only slightly better absorbed than from alperujo without β-CD. A previous study showed that hydroxypropyl-β-CD improved naringenin absorption through the gut epithelium by 11-fold [40]. Lee et al. (2007) also increased the absorption of soy isoflavones in rats by 126% following their binding to β-CD [41]. The main recognized advantage of β-CD is to increase the apparent water solubility and dissolution rate of phenolic compounds [17,41]. The lower improvement observed with the olive phenols in this study could be due to their higher water solubility and lower affinity for β-CD than flavonoids.

Our estimation of the phenolic compound in vitro bioavailability, expressed as % bioaccessibility × % absorption by Caco-2 cells, takes into account both the digestion and the absorption processes, and is thus more reliable than the common (over)estimations based on pure compound absorption. The in vitro bioavailability for each phenolic compound was not significantly different whether alperujo was administered with or without β-CD, which may be due to the low amounts of phenolic compounds recovered in the basolateral chamber, compared to the initial amounts brought by the meal. However, this calculation confirmed that *p*-Cm was the most bioavailable compound among alperujo phenols, followed by Tyr and CA (under FA form). These differences in bioavailability may be due to differences in intrinsic properties (polarity, solubility), but also to competition for absorption between phenolic compounds, including with other more minor alperujo components [42].

## 5. Conclusions

When ingested within a meal, β-CD has no significant effect on the bioaccessibility of phenolic compounds from alperujo. The bioavailability of the phenolic compounds (and their metabolites) from alperujo, defined as a ratio between their basolateral concentration and their initial concentration in the meal, was as follows: *p*-Cm > Tyr > CA. The moderate bioavailability of these olive phenols (8.9 to 37.2%) could result from their low water solubility, their high metabolism in the intestine, and the possible competitions for absorption between them and other nutrients or alperujo compounds. Interestingly, β-CD was able to locally increase the absorption of Tyr, *p*-Cm, and CA through the intestinal barrier (*p* < 0.05). However, β-CD did not modify the overall in vitro bioavailability of Tyr, *p*-Cm, and CA. β-CD thus appears as a versatile tool to extract phenolic compounds from plant material and improve their storage [43,44], and can be used in either supplements or food products without altering their bioavailability.

## Figures and Tables

**Table 1:** Bioavailability of phenolic compounds in Caco-2 cells as percentage of their initial apical amount.

Samples	Tyr	*p*-Cm	FA
Alperujo	22.9 ± 0.7 ^a^	40.2 ± 4.1 ^a^	18.8 ± 1.0 ^a^
Alperujo-CD	25.2 ± 1.3 ^b^	48.7 ± 2.0 ^b^	23.4 ± 2.2 ^b^

Values are expressed as mean ± SD (*n* = 4). Different letters indicate a significant difference according to Tukey test (*p* ≤ 0.05) between the alperujo and alperujo-CD conditions for each phenolic compound.

**Table 2:** Bioavailability of the phenolic compounds in Caco-2 cells as percentage of their initial amount in the test meal before in vitro digestion.

Samples	Tyr	*p*-Cm	FA
Alperujo	16.2 ± 0.6 ^a^	32.0 ± 3.2 ^a^	8.9 ± 0.5 ^a^
Alperujo-CD	16.8 ± 0.5 ^a^	37.2 ± 3.2 ^a^	10.0 ± 1.0 ^a^

Values are expressed as mean ± SD (*n* = 4). Different letters indicate a significant difference according to Tukey test (*p* ≤ 0.05) between the alperujo and alperujo-CD conditions for each phenolic compound.
